# Clinical Spectrum and Medical Comorbidities in Tuberculosis: A Hospital-Based Study in Northeast India

**DOI:** 10.7759/cureus.10580

**Published:** 2020-09-21

**Authors:** Prasanta Bhattacharya, Kishore Talukdar, Bhupen Barman, Md Jamil, Pranjal Phukan, Habung Mobing, Gwenette War, Phibakordor L Nonglait, Subrahmanya Murti, Konthoujam Prithviraj, Bandi Sangma

**Affiliations:** 1 Department of General Medicine, North Eastern Indira Gandhi Regional Institute of Health and Medical Sciences (NEIGRIHMS), Shillong, IND; 2 Department of Radiology, North Eastern Indira Gandhi Regional Institute of Health and Medical Sciences (NEIGRIHMS), Shillong, IND; 3 Department of Cardiology, Smt. Nathiba Hargovandas Lakhmichand (NHL) Municipal Medical College (NHLMMC), Ahmedabad, IND; 4 Department of Chest & Tuberculosis, North Eastern Indira Gandhi Regional Institute of Health and Medical Sciences (NEIGRIHMS), Shillong, IND

**Keywords:** pulmonary tuberculosis (ptb), extrapulmonary tuberculosis (eptb), diabetes mellitus (dm), anti tubercular therapy (att)

## Abstract

Background

Tuberculosis (TB) is one of the most common infectious diseases and is commonly associated with comorbidities. However, data regarding TB and comorbidities are lacking from northeast India. The aim of the study is to see the clinical spectrum of TB and the frequency of comorbidities.

Methods

This was a prospective observational study of all hospitalized TB patients between January 2016 and June 2017 who were selected by consecutive sampling. Data were analyzed using SPSS v. 17.0 (IBM Corp., Armonk, NY), and a p-value of <0.05 was considered significant.

Results

Of the 173 patients selected, the mean age was 41.05±17.04 years with a male:female ratio of 4.27:1. Pulmonary TB (PTB) was found in 43.94%, extra-pulmonary TB (EPTB) in 52.02%, and disseminated TB in 4.04%. Fever (61.27%) was the most common presentation, followed by cough (54.33%) and breathlessness (32.94%). Of the 76 patients with PTB and seven with disseminated TB, making a total of 83 patients, 56 (67.4%) were sputum positive. Out of 90 patients suffering from EPTB, pleural effusion (53.33%) was the commonest type of EPTB, followed by central nervous system (CNS) tuberculosis (26.66%) and abdominal tuberculosis (8.88%). Comorbidities were present in 53.17% of the patients, of which diabetes mellitus (DM) (26.58%) and hypertension (17.34%) were the most common. Comorbid conditions were significantly higher in PTB than EPTB (51 of 83 vs. 41 of 90, p<0.05). Mean glycated hemoglobin (HbA1c) was significantly higher in PTB as compared to EPTB (8.74±2.04 vs. 7.58±0.29, p<0.05).

Conclusion

Comorbidities, particularly DM, were present in half of the patients, mostly in PTB than EPTB, with glycemic control being significantly poorer in PTB patients.

## Introduction

India accounts for one-fourth of the global TB burden of tuberculosis (TB) in the world, with an estimated 2.8 million cases in 2015 [1]. About 40% of the Indian population is infected with TB, the majority having a latent infection, which can potentially flare up into active disease. While extra-pulmonary tuberculosis (EPTB) accounts for a quarter of the annual incidence of TB globally, the overall literature available regarding the spectrum of EPTB is limited.

Most countries with a high TB burden also face a concomitant burden of associated medical comorbidities. These include liver diseases, chronic kidney diseases (CKD), diabetes mellitus (DM), cardiovascular diseases, connective tissue disorders, chronic obstructive pulmonary disease, and so on. Such medical comorbidities interact with TB at multiple levels. On one hand, these may aggravate the tubercular process from latent to active TB or even to disseminated forms, may cause diagnostic challenges, and lead to ineffective treatment. On the other hand, these comorbidities may restrict the use of some potent anti-tubercular drugs. Furthermore, TB itself can aggravate the comorbidities or hinder in the diagnosis and/or optimum management of such comorbidities.

Published data on TB and associated comorbidities from the North-Eastern states of India are limited. Out of a total of 14,24,771 newly notified cases of TB in India in 2015, 53,427 cases were from the northeastern states, with Assam accounting for the majority with 36724 cases followed by Meghalaya (3,934 cases) and the least from Sikkim (1,463 cases) [1]. With this background, this study was planned to study the clinical spectrum of TB and the association of comorbidities in patients attending a tertiary care center in northeast India, which caters to most of the patients in the state.

## Materials and methods

This hospital-based prospective observational study was conducted for all cases of tuberculosis admitted to the department of general medicine between January 1, 2016, and December 31, 2016. During the study period, 654 tuberculosis patients got registered at the DOTS (directly observed treatment, short-course) center for antitubercular therapy (ATT). A total of 173 patients admitted during the study period in the department of general medicine were included in the study group by the consecutive sampling method. Ethical clearance was taken from the Institutional Ethics Committee prior to the commencement of the study. The diagnosis of TB was made in all the cases fulfilling the following criteria:

· Sputum positive TB

· Sputum negative TB but conventional chest radiography suggestive of TB

· Fluid cytology suggestive of TB

· Fine needle aspiration cytology (FNAC)/biopsy confirmation of TB

· Computed tomography (CT) scan (contrast or non-contrast)/magnetic resonance imaging (MRI) scan suggestive of TB

Information was collected after obtaining valid informed consent. The socio-demographical, clinical, and laboratory parameters and outcomes were assessed. The demographic data included were age, sex, ethnic and occupational background. Clinical data comprised symptoms related to the primary etiology of tuberculosis and its predisposing factors and/or associated comorbidities. Laboratory investigations included complete hemogram, sputum analysis for acid-fast bacilli (AFB), blood sugar estimation, blood urea, serum creatinine, serum electrolytes, liver function tests, coagulation profile, and routine urine analysis. Wherever necessary, fluid cytology, FNAC, biopsy, urine for AFB, chest X-ray, ultrasonography (USG) of the abdomen, and other ancillary biochemical and radiological investigations were done.

Statistical analysis

To describe the characteristics of the study population, Microsoft Excel 2010 (Microsoft Corporation, Redmond, Washington) was used. SPSS Statistics version 17.0 (IBM Corp., Armonk, NY) was used to perform the statistical analysis. Continuous data were presented as mean (with SD) and the categorical data were expressed as count (with percentage). Analyses of categorical variables were performed by the chi-square test. A p-value of <0.05 was considered significant.

## Results

The case group consisted of 173 patients (age = 41.05±17.04 years) of which the majority were males (80.93%), with a male to female ratio of 4.27:1. The most common age group was 18-30 years for both males and females. Out of the 173, 76 (43.94%) patients were pulmonary tuberculosis (PTB), 90 (52.02%) patients were extra-pulmonary tuberculosis (EPTB), and the remaining seven (4.04%) were disseminated tuberculosis. All cases of disseminated tuberculosis in the present study had pulmonary involvement. For the purposes of comparison between PTB and EPTB, disseminated cases were considered in the PTB bracket. Among seven cases of disseminated tuberculosis, five had comorbid HIV infection, with all patients having a cluster of differentiation 4 (CD4) count of less than 50 cells/µL. Both PTB and EPTB were more common in males as compared to females. Further, among 138 male patients, PTB (71 of 138, i.e. 51.44% was more common than EPTB, but among 35 female patients, EPTB (28 of 35 i.e. 80%) was more common than PTB. The mean age in PTB patients (42.86±16.2 years) was higher than in EPTB patients (38.94±17.45 years); the difference being statistically insignificant. The average duration of hospital stay in patients with TB was 7.59±4.72 days. The mean duration of hospitalization in PTB, EPTB, and disseminated TB were 6.2±4.75 days, 6.96±4.8 days, and 13.28±5.12 days, respectively.

The most common symptomatic presentation among our patients was low-grade fever, with an evening rise of temperature (61.27%) followed by cough (54.33%), breathlessness (32.94%), and altered sensorium (17.64%) (Table 1). The most common predisposing behavioral risks observed in this study were smoking (59.53%), alcohol abuse (56.64%), and tobacco (42.19%). Alcohol use was significantly higher in PTB as compared to EPTB (p<0.05). Nineteen point sixty-five percent (19.65%) of the patients did not have any such predisposing factor (Table 2). Out of 90 patients suffering from EPTB, tubercular pleural effusion (53.33%) was the commonest type of EPTB, followed by CNS tuberculosis (26.66%) and abdominal tuberculosis (8.88%) (Table 3).

**Table 1:** Showing presenting symptoms of patients with tuberculosis

Presenting symptom	Number of patients (%)			
	PTB (n=76)	EPTB (n=90)	Disseminated=7	Total (n=173)
Abdominal distension	1	8	-	9 (5.20)
Altered sensorium	1	22	4	27 (17.64)
Breathlessness	20	35	2	57 (32.94)
Chest discomfort	10	13	-	23 (13.29)
Chest pain	7	8	-	15 (8.67)
Chronic diarrhea	0	2	1	3 (1.73)
Cough	63	31	-	94 (54.33)
Fever	57	49	-	106 (61.27)
Generalized weakness	10	7	-	17 (9.82)
Hemoptysis	17	0	-	17 (9.82)
Jaundice	1	11	-	12 (6.93)
Oliguria	1	5	-	6 (3.46)
Weight loss	3	-	3	8 (4.62)

**Table 2:** Showing behavioral risk factor in patients with tuberculosis IV: intravenous

Behavioral risk	PTB (n=76)	EPTB (n=90)	Disseminated= (n=7)	Total (n=173)	P-value (PTB+disseminated vs EPTB)
Smoking	50	51	2	103 (59.53)	p>0.05
Alcohol consumption	53	40	5	98 (56.64)	P<0.05
Tobacco chewing	30	38	5	73 (42.19)	P>0.05
IV drug users	1	1	2	4 (2.31)	P>0.05
High risk sexual history	2	0	2	4(2.31)	P<0.05
None	15	18	1	34 (19.65)	P>0.05

**Table 3:** Showing the incidence of different types of EPTB CNS: central nervous system; EPTB: extra-pulmonary tuberculosis

Type of EPTB	Number of patient (%)		
	Male	Female	Total
Abdominal tuberculosis	5	3	8 (8.88)
CNS tuberculosis	16	8	24 (26.66)
Pericardial effusion	1	2	3 (3.33)
Pleural effusion	33	15	48 (53.33)
Spine tuberculosis	1	1	2 (2.73)
Tubercular lymphadenopathy	3	2	5 (5.07)
Total	59	31	90 (100)

In the present study, various medical comorbidities were present in 53.17% of the patients, of which diabetes mellitus (26.58%) and hypertension (17.34%) were the most common. Comorbid conditions were significantly higher in PTB than EPTB (51 of 83 vs. 41 of 90, p<0.05). Also, the mean age of patients with comorbidity was significantly higher than those without comorbidities (46.12±18.02 vs. 34.42±16.51, p<0.05). All the comorbid conditions, except for ischemic heart disease, were more common in PTB as compared to EPTB, however, diabetes mellitus and chronic kidney disease were significantly higher in PTB as compared to EPTB (Table 4). Mean HbA1c was significantly higher in PTB as compared to EPTB (8.74±2.04 vs. 7.58±0.29, p<0.05).

**Table 4:** Showing distribution of comorbid medical conditions *all HIV comorbid patients are disseminated tuberculosis PTB: pulmonary tuberculosis; EPTB: extra-pulmonary tuberculosis; HIV: human immunodeficiency virus

Comorbidity	PTB + disseminated n=83	EPTB N=90	Total (% of n=173)	Mean age (years)	P-value
Diabetes mellitus	35	11	46 (26.58)	45.96	P<0.05
Hypertension	16	14	27 (17.34)	54	p>0.05
Ischemic heart disease	1	3	4 (2.31)	48.5	p>0.05
Chronic liver disease	9	8	17 (9.82)	44.33	p>0.05
Chronic kidney disease	5	1	6 (3.46)	44.5	P<0.05
Chronic obstructive pulmonary disease	3	3	6 (3.46)	57.67	p>0.05
Connective tissue disorder	2	0	2 (1.15)	25	p>0.05
Hypothyroidism	4	3	6 (3.46)	43.5	p>0.05
HIV*	5	0	5 (2.89)	29.4	-
	Overall PTB + disseminated	Overall EPTB			
Comorbidity	51	41	92 (53.17)	46.12±18.02	
No comorbidity	32	49	81 (46.83)	34.42±16.51	
P-value			0.03	0.04	
Grand total	83	90	173 (100)		

Of the 76 (43.94 %) patients suffering from pulmonary tuberculosis and seven (4.04%) with disseminated tuberculosis, making a total of 83 patients, 56 (67.4%) were sputum positive, the rest being sputum negative. Of the seven patients with disseminated tuberculosis, only two (28.57%) were sputum positive - one male and female, respectively. On comparing sputum-positive patients (56) to sputum-negative patients (27), pulmonary tuberculosis, comorbid conditions (36 of 56 vs. 18 of 27, p>0.05), presence of diabetes (20 of 56 vs. 15 of 27, p>0.05), and mean HbA1c(8.95±1.6 vs. 8.16±3.41, p>0.05) were higher in sputum-positive PTB, but the results were not statistically significant (Table 5).

**Table 5:** Showing comparisons between sputum-positive and sputum-negative PTB PTB: pulmonary tuberculosis

Type of PTB	Number of patients (%)			P-value
	Comorbidity	No comorbidity	Total	
Sputum positive (SP)	36	20	56	0.07
Sputum negative (SN)	18	9	27	
Total	54	29	83	-
Type of PTB	Number of patients (%)			P-value
	Diabetes	No diabetes	Total	
SP	20	36	56	0.08
SN	15	12	27	
Total	35	48	83	-

ATT was given to all patients, of which 135 (78.03%) were given Category I ATT (61 (45.18%) had PTB and 74 (54.82%) had EPTB) and the remaining 38 patients were given Category II ATT (22 (57.89%) had PTB and 16 (42.01%) had EPTB). On a comparison between retreatment cases (i.e. those on Category II) as compared to new cases, the proportion of PTB was significantly higher in the re-treatment group (P<0.05).

## Discussion

In the present study, the majority of patients were male (80.93%) with a male to female ratio of 4.27:1. The mean age was 41.05±17.04 years with the most common age group being 18-30 years. Worldwide, including the latest Indian data, males have a higher prevalence of tuberculosis [1]. The exact reason for the same is still a subject of active research [2]. The most common age group for tuberculosis in India is the third decade as per the latest Revised National TB Control Program (RNTCP) data. This is also reflected in a large systematic review, as well as a large population-based study from Europe [3-4]. However, there is a rising trend of tuberculosis in the elderly, as reflected in national and international data [5]. In the present study, EPTB was higher in females than males as against the norm of male preponderance. The same trend of female preponderance in EPTB, particularly pleural effusion, was also shown in a recent gap analysis of south Asian studies on EPTB [6].

Among predisposing behavioral risks, smoking (59.53%) was most commonly found in patients with tuberculosis followed by alcohol consumption (56.64%) and tobacco chewing (42.19%). Smoking has long been linked to tuberculosis. The first strong relation was proposed in a systematic review in 2005, with evidence becoming stronger from a recent population-based study from China that showed a two-fold risk of pulmonary tuberculosis with smoking [7-8]. Alcohol use has also been associated with tuberculosis with a large systematic review suggesting a causal association [9]. A recent meta-analysis has shown that alcohol use led to 2.35 deaths and 22.02 incident cases per 1,00,000 people from tuberculosis in 2014 [10].

In the present study, 27 out of 83 (32.53%) pulmonary tuberculosis patients were sputum negative. Sputum-negative pulmonary tuberculosis is a diagnostic and treatment challenge wherein patients have radiologically active lesions with no evidence of acid-fast bacilli in the sputum. These patients may be further culture positive or negative. Sputum-negative pulmonary tuberculosis has been linked to a high incidence of HIV coinfection in a population-based study from Brazil [11]. Further, in a population-based study from the Netherlands, patients with smear-negative, culture-positive tuberculosis were responsible for 13% of TB transmission [12]. This highlights the importance of this subset of TB patients. In the present study, a majority (68.63%) of all extrapulmonary cases were tubercular pleural effusions followed by abdominal tuberculosis. This is in conformity with most Indian data as from Karnataka and Himachal Pradesh [13-14]. However, a study from Bhopal found a higher incidence of TB lymphadenitis as compared to a pleural effusion [15].

In the present study, various medical comorbidities were present in 53.17% of the patients, of which diabetes mellitus (26.58%) and hypertension (17.34%) were the most common. Comorbid conditions had a significantly higher association with PTB than EPTB. Diabetes mellitus (DM) and chronic kidney disease (CKD) had a significantly higher association with PTB than EPTB. Mean HbA1c was also significantly higher in PTB as compared to EPTB. Diabetes and tuberculosis have been well-recognized as intersecting epidemics. The presence of diabetes has been shown as an independent risk factor for death in TB in a large retrospective cohort study [16]. Tuberculosis and chronic kidney disease are now recognized as syndetic [17]. Tuberculosis has shown both to be a risk factor for chronic kidney disease, as well as being more prevalent in the community with chronic kidney disease from an Indian study [18-19].

Chronic liver disease (CLD) and cardiovascular disease (CVD) were also more common in PTB than EPTB, with the difference being insignificant. Liver cirrhosis has also shown to be a risk factor for TB. In a large nationwide cohort study from Taiwan, 957 of 41,076 cirrhotic patients developed TB, which was significantly higher than the 955 of 204,244 noncirrhotic patients (P < 0.001) [20]. Tuberculosis has been associated with an increased CVD risk. Another nationwide cohort study with a three-year follow-up from Taiwan has shown that the presence of TB confers a higher risk of ischemic stroke [21]. Also, another study has shown that latent TB infection is independently associated with a higher risk of acute myocardial infarction [22].

Limitation

As the study was done in a tertiary care center, where the types of patients were mostly in the moderate to severe category with multiple comorbidities, there are chances of an inherent bias being present.

## Conclusions

Tuberculosis was more common in young males. Among PTB patients, sputum-positive cases were twice as common as sputum-negative cases while pleural effusion was the most common type of EPTB. Medical comorbidities were found in nearly half the patients, being significantly more common among PTB than EPTB. Diabetes mellitus was the most common co-morbidity, which was more common and associated with significantly poorer glycaemic control in PTB as compared to EPTB. A significantly higher proportion of retreatment cases had PTB.
